# Implications of not matching to a first-choice discipline: a family medicine perspective

**Published:** 2017-06-30

**Authors:** Wayne Woloschuk, Douglas Myhre, James Dickinson, Shelley Ross

**Affiliations:** 1 University of Calgary, Alberta, Canada; 2 University of Alberta, Alberta, Canada

## Abstract

**Background:**

Family medicine is often selected as an alternate career choice by medical students who do not match to their first choice discipline. Consequently, family medicine residency programs accept and train some residents who prepared for and intended a career in another specialty. The implications of this warrant investigation.

**Methods:**

Graduates (2006–2011) of Albertan family medicine residency programs were surveyed to examine differences between physicians who indicated family medicine was their first choice discipline and those who indicated that it was not their first choice. Survey questions targeted practice location, preparedness for practice, perceptions of family medicine, lifestyle satisfaction, and well-being. Principal components analysis was used to examine the factor structure of our survey items and ANOVA and Chi square were used to compare mean scores and proportions, respectively.

**Results:**

The overall response rate was 47.2% (307/651). Most (263) respondents reported that family medicine was their first choice discipline (yes-group); 42 respondents indicated that it was not (no-group) and two did not answer. The two groups were similar demographically. The no-group reported significantly lower mean scores on perceptions of family medicine. There were no significant differences between the two groups in their preparedness for practice and measures of lifestyle satisfaction and well-being.

**Conclusion:**

Irrespective of their perceptions of the discipline, the respondents who did not match to their first choice discipline found family medicine to be a viable career option.

## Introduction

The duration of medical school in Canada is either three or four years. Additional training at the postgraduate level involves at least two more years of training, depending on the discipline. To enter postgraduate training, students must compete for residency positions. They do this through the Canadian Resident Matching Service (CaRMS) which attempts to place students in their top choice of residency training programs. The recent 2016 CaRMS report^1^ indicates that, in the first iteration, 88.6% of students were successful in matching to their first choice discipline. Approximately 11% of students matched to a discipline that was a second or lower ranked choice. A match rate of approximately 8–10% to a second choice discipline or lower has been consistent for about the past decade.^1^ Although the process of selecting a suitable residency program is multifaceted and complex,^2^,^3^ each year a small but not insignificant percentage of medical students match to a discipline that is not their first choice. Most of these students are allocated to family medicine that is used by some graduating students as a “back up” discipline. However, students whose primary career choice is family medicine possess attributes that differ from those who prefer other medical careers^4^ and costs associated with residents who go on to experience difficulty can be significant.^5^ Therefore consequences of training and practicing in a discipline other than the one chosen first should be investigated.

The objective of this study was to look for systematic differences in demographics, practice preparedness, perceptions of the discipline, lifestyle satisfaction, and well-being between family physicians who entered family medicine as their first choice and those for whom it was not their first choice.

## Methods

This was a collaborative study carried out by the Departments of Family Medicine (DoFM) at the University of Calgary (UC) and the University of Alberta (UA). Data were collected using a self-administered questionnaire that was pilot tested prior to distribution. Contact information was available for 651 (92%) graduates. Each DoFM mailed the questionnaire in July, 2013 to their own graduates who exited the program (396 UA; 255 UC) during the years 2006–2011. Following the initial distribution of the questionnaire, non-respondents were contacted a maximum of five times. The survey remained open until December of 2014. Completed questionnaires were returned by prepaid post. Each questionnaire was numerically coded to maintain confidentiality. In addition to demographics, the questionnaire addressed: a) medical education, b) career history, c) intimidation, harassment, and discrimination, d) physician wellness, e) family medicine program evaluation, and f) perceptions about family medicine. In the section on medical education, the survey asked participants, “Was the discipline of family medicine your first choice for a residency program?” Responses to this item (yes/no) served as the independent variable. The dependent variables included the demographics of sex, age, and practice location (rural vs non-rural), plus items about preparedness for practice, perceptions about family medicine, and levels of lifestyle satisfaction and well-being. Participants were asked to rate their preparedness for practice (4-point scale) after completing the family medicine residency program. Response options were Very Unprepared, Unprepared, Prepared, and Very Prepared. Perceptions about family medicine comprised seven items to which respondents reported agreement that ranged from Strongly Disagree (1) to Strongly Agree (5). Lifestyle Satisfaction consisted of nine items to which respondents reported satisfaction that ranged from Very Dissatisfied (1) to Very Satisfied (5). Well-being comprised three items with response options that ranged from Poor (1) to Excellent (5). We previously created the lifestyle satisfaction items to survey an earlier cohort of family medicine graduates. Items measuring perceptions of family medicine and well-being were new and developed specifically for this survey. The study was approved by the Human Research Ethics Board at UA and the Conjoint Health Ethics Board at UC.

### Data analysis

To understand the factor structure of the scales that assessed perceptions about family medicine, lifestyle satisfaction, and well-being, a principal components analysis (PCA) with varimax rotation was performed on each one. Factors that possessed an Eigenvalue greater than one were retained and a minimum factor loading of 0.50 was set. Cronbach’s alpha was used to estimate the internal consistency of the factors.

Pearson correlation coefficient was used to examine relationships. Chi square was used to compare proportions while ANOVA, a *t*-test equivalent for two-group analyses, was employed to compare mean scores. Cohen’s *d* was used to report effect size. Data were analyzed using Statistica 12.^6^

## Results

The overall response rate was 47.2% (307/651) with response rates of 46.2% (183/396) and 48.6% (124/255) for the UA and UC graduates respectively. When survey responses are low, describing the characteristics of non-respondents becomes important.^7^ Of the respondents, 59.6% (183/307) were UA graduates while 61.9% (213/344) of non-respondents were UA graduates. Of the respondents, 40.4% (124/307) were UC graduates while 38.1% (131/344) of non-respondents were UC graduates, p = 0.55. Of the respondents, 61.8% (188/304) were female compared to 57.4% (197/343) of non-respondents. Of the respondents, 38.2% (116/304) were male while 42.6% (146/343) of non-respondents were male, p = 0.25. Two respondents did not indicate sex and one respondent was transgendered. The sex of one non-respondent was unknown.

Most (263) respondents reported that family medicine was their first choice discipline (yes-group) while 42 respondents indicated that it was not their first choice (no-group). Two respondents did not answer this question. The two groups did not differ on demographic features of age, sex and practice location or preparedness for practice (Table 1).

The factor analysis of the seven items that addressed perceptions of family medicine produced both two and three factor solutions. Therefore, using the principle of Occam’s razor, we adopted the two factor model (Table 2) which was more interpretable and accounted for 55.9% of the variance.

Based on the nature of the items and respective loadings, factor 1 was labelled *family medicine identity* and factor 2 was labelled *external view of family medicine* and accounted for 38.4% and 17.5% of the variance, respectively. The one item on each factor that loaded negatively was reverse scored so that a higher score on all items reflected a positive view. The reliability of factor 1 was 0.64 and of factor 2 was 0.62. A mean score on each factor was calculated for all respondents.

The factor analysis of the nine items that addressed lifestyle satisfaction initially produced a two factor model. However, we eliminated the continuing medical education item (satisfaction with CME opportunities) as it cross-loaded on both factors leaving the locum item (satisfaction with your locum availability) alone as the second factor. To avoid a single item factor, we selected the remaining seven items to represent the lifestyle satisfaction factor (Table 3). The seven items accounted for 52% of the variance. The reliability of data produced by the seven item scale was estimated at 0.89. A mean score for each respondent was calculated.

The factor analysis of the well-being items (Table 4) indicated that the scale was unidimensional. Consequently, a mean well-being score was calculated for each respondent using all three items which explained 81% of the total variance. The scale reliability was estimated to be 0.89. Well-being and lifestyle satisfaction scores were moderately correlated (r=0.45, p < .05).

The mean scores for the two groups (yes and no) on each dependent measure are presented in Table 5. Physicians who entered family medicine as a first-choice discipline (yes group) had higher mean scores on two measures, family medicine identity (4.61 vs. 4.12; p=.000001) and external view of family medicine (3.67 vs. 3.40; p=.016) with statistically significant differences. The mean scores for the yes-group and no-group on measures of lifestyle satisfaction and well-being did not differ.

## Discussion

For this cohort of graduates two to seven years following program completion, the yes-group and no-group respondents were similar in age, sex, and practice location. Approximately 95% of both groups reported being prepared or very prepared for practice at the time of exit from post-graduate training. This suggests that the two residency programs were able to prepare residents for practice regardless of their incoming disposition and prior learning. That is, just a few months prior to beginning family medicine residency some participants in this study were hoping to train in other disciplines and most likely had shaped their medical education by selecting electives related to their chosen fields. It is notable that the trainees who expected to be elsewhere reported the same level of preparedness for practice as their peers who selected family medicine first.

Our measure of lifestyle satisfaction comprised seven items that included professional life, family life, community life, work-life balance, personal health, income, and practice arrangements. The mean score on lifestyle satisfaction for both groups was similar and high (≥ 4.0/5). This finding is important as physician job satisfaction^8^–^10^ appears to be related to quality of patient care.

Nearly twenty years ago the Canadian Medical Association (CMA) recognized the importance of physician well-being by releasing the CMA Policy on Physician Health and Well-being.^11^ We measured well-being using the total score to three items reflecting current levels of personal and professional well-being plus general health. Well-being was rated as very good for both groups. This finding is reassuring because previous research reports that physician wellness or well-being may affect patient care^12^–^14^ and poor physician health may lead to medical error.^15^

Our investigation of perceptions of family medicine produced a primary factor that we labelled as family medicine identity. Identity may be theorized as an “ongoing process that encompasses the sense of self created through social interactions.”^2^ Identity formation by physicians begins in medical school where they assume the professional norms and values and learn the discourse of the profession.^2^ Facilitating the development of professional identity should be central to the medical education process.^16^–^18^ In this study, both groups reported high family medicine identity scores. However, the mean score for the yes-group was higher than the mean score reported by the no-group (4.61 vs. 4.12). Those in the no-group showed a positive, albeit lower, level of identity with the profession, which may not be surprising considering that they had anticipated practicing in another discipline. Switching from another discipline to family medicine and reformulating a professional identity is a process that entails personal negotiation and may be challenging.^19^ We do not know whether the no-group encountered difficulties in redeveloping their identity as a family physician and if a lower identity with the discipline affects their quality of care but both of these questions are worthy of further study. To understand better the adjustment to family medicine future research should interview physicians who switched to learn about the transition process including whether they shaped their practice towards their preferred discipline.

In studying the perceptions of family medicine, we also observed a secondary factor that we labelled external view of family medicine. One’s external view of the discipline or perceived image reflects how physicians believe others (patients, colleagues, and government) value them. Although both groups reported scores in the moderate range for external view of family medicine those in the yes-group reported a higher mean score (3.67 vs. 3.40). That is, their perceived image is more positive than the perceived image of the no-group. Perceived image is important because the way professionals identify with their profession may be shaped by how they believe others view them.^2^ The lower score for the no-group may reflect a variety of complex reasons including their own personal perceptions of the discipline. It was also noted that the perceived image of family physicians by both groups was in the moderate range and below a level that would indicate a strong external endorsement of the discipline. This may be a result of “badmouthing” directed at the discipline by others 20,21 and from the perceived lack of prestige reinforced throughout medical training that connects medical expertise to specialized knowledge^2^. It may also reflect differentials in economic rewards.

### Limitations

This study has a number of limitations. It is possible that other more objective measures may find additional differences between the two groups which our questionnaire did not address. Additionally, the cross-sectional nature of our study attempted to capture self-report data at a single point in time. Since time from graduation and survey distribution varied between two and seven years, the influence of recall bias cannot be dismissed. Furthermore, our overall response was low.

Consequently, we compared the proportion of respondents and non-respondents on two variables (school of graduation and sex) and found no differences. Although this suggests no bias on these two variables, we were unable to examine other potentially important variables so the presence of some non-response bias is possible. Respondents who did not choose family medicine as their first choice are not homogeneous in the routes taken to enter a family medicine residency program. However, we placed all respondents in the no-group together and are unsure whether doing so affected the findings. It should also be noted that the low number of no-group respondents is not due to a worse response rate by physicians whose first choice was not family medicine. There is an imbalance in the size of the groups because the majority of family physician respondents selected family medicine first, and over time the selection rate of family medicine as first choice has been increasing. Our 42 no-group respondents represent 13.7% of total respondents which is similar to the percentage (8–10%) of students who do not get their first choice discipline in the CaRMS match. That is, most family physicians are in family medicine because that was their first choice discipline. The factor analyses of our outcome measures provided evidence of construct validity. The reliability of data generated by the various measures ranged from moderate to high. We expected lifestyle satisfaction and well-being to measure different but related constructs. This premise was supported by the moderate correlation between lifestyle satisfaction and well-being scores.

### Conclusion

The identity with family medicine of those who planned to enter another discipline prior to the CaRMS match was positive, but not as strong as those who entered family medicine as a first choice. Their perception of how colleagues in other disciplines and the general public view them was also lower. Most importantly, no other differences were found between the two groups on dimensions that we examined including demographics, preparedness for practice, lifestyle satisfaction, and well-being. Given the current residency match process and needs of society, there will always be physicians practicing in a discipline that was not their first choice and most of these will be family physicians. Although the family physicians in this study who did not match to their first choice discipline reported lower levels of both identity and perceived image of family medicine, it is reassuring that they graduated feeling prepared for practice and their adjustment to family medicine afforded them a lifestyle and level of well-being comparable to their colleagues.

## Figures and Tables

**Table 1:** Comparison of FM first choice-Yes and FM first choice-No groups on demographics and preparedness for practice.

Demographic	FM first choice-Yes	FM first choice-No	Significance
Age (Mean/SD)	38.63 (6.96)	37.10 (4.92)	*F =* 1.85*; P* = 0.18
N (%) Female	162 (62.3)	26 (61.9)	*X*^2^ *=* 0.00*; P* = 0.96
N (%) Rural Practice (pop < 25,000)	63 (25.2)	5 (12.2)	*X*^2^ *=* 3.33*; P* = 0.07
N (%) Prepared/Very Prepared for Practice	244 (94.6)	40 (95.2)	*X*^2^ *=* 0.04*; P* = 0.85

**Table 2:** Perceptions about family medicine factors, items and loadings.

Factor 1: Family Medicine Identity	Factor loading
I am proud to be a family physician	0.78
Family physicians make a valuable contribution that is different from other specialists	0.71
I would prefer to be in another medical specialty	−0.75
**Factor 2: External view of Family Medicine**	
Government perceives family medicine as essential to the health care system in Canada	0.73
Patients recognize the value of family medicine	0.71
Patients believe that family physicians provide value above and beyond referring to other types of specialists	0.68
I have found that other medical specialists have little respect for the expertise of family physicians	−0.55

**Table 3:** Lifestyle satisfaction items and factor loadings

Satisfaction with:	Factor loading
Professional life	0.83
Family life	0.72
Practice arrangements	0.81
Income	0.75
Community life	0.75
Work-life balance	0.66
Health status	0.73

**Table 4:** Well-being items and factor loadings

Items	Factor loading
Your personal well-being	0.94
Your professional well-being	0.89
Your general health status	0.88

**Table 5:** Comparison of mean scores (SD) on FM identify, external view of FM, lifestyle satisfaction, and well-being according to FM first choice-Yes vs. FM first choice-No.

Measure 5-point scale	FM first choice-Yes	FM first choice-No	Significance
Family medicine identity	4.61 (0.53)	4.12 ( 0.81)	*F =* 26.27; *P* = .000001 *d* = .85
External view of family medicine	3.67 (0.67)	3.40 (0.75)	*F =* 5.90; *P* = .016 *d* = .40
Lifestyle satisfaction	4.21 (0.73)	4.02 (0.70)	*F =* 2.49; *P* = .12
Well-being	4.02 (0.78)	3.93 (0.74)	*F =* 0.48; *P* = .49
